# Optimal transport analysis of high-dimensional flow cytometry data in immuno-oncology

**DOI:** 10.3389/fimmu.2026.1856896

**Published:** 2026-07-28

**Authors:** Abida Sanjana Shemonti, Justin C. Wang, Albert D. Donnenberg, Bartek Rajwa, Patrick L. Wagner, David L. Bartlett, Bosko Popov, Evan T. Alicuben, Vera S. Donnenberg

**Affiliations:** 1 Miftek Corporation, West Lafayette, IN, United States; 2 University of Pittsburgh School of Medicine, Pittsburgh, PA, United States; 3 Allegheny Health Network Research Institute, Pittsburgh, PA, United States; 4 Department of Medicine, Drexel University College of Medicine, Philadelphia, PA, United States; 5 Bindley Bioscience Center, Purdue University, West Lafayette, IN, United States; 6 Allegheny Health Network Cancer Institute, Division of Surgical Oncology, Pittsburgh, PA, United States; 7 University of Pittsburgh Medical Center (UPMC) Hillman Cancer Center, Pittsburgh, PA, United States; 8 Department of Cardiothoracic Surgery, University of Pittsburgh School of Medicine, Pittsburgh, PA, United States

**Keywords:** clinical trial endpoints, immuno-oncology, malignant pleural effusion, omics, optimal transport, peritoneal ascites, Sinkhorn algorithm

## Abstract

**Introduction:**

Advances in single-cell and spatial profiling have enabled detailed characterization of heterogeneous samples, but analyzing this data remains challenging in settings involving multiple comparisons. While tools like UMAP and t-SNE are valuable for visualization, their stochastic, parameter-sensitive nature limits their use in longitudinal comparisons, treatment group analysis, and multicenter trials. Although OT was first described in the 19th century, the Sinkhorn algorithm makes it computationally tractable for high-dimensional data. By directly comparing distributions of cellular states, OT provides reproducible measures of change in high-dimensional space. This framework is amenable to integration with machine learning, including deep generative models.

**Methods:**

OT was applied to longitudinal data from a phase I trial of tocilizumab for cavitary malignancies (NCT 06016179). The current implementation makes use of expert-guided phenotypic population definitions and their relationships. An OT-based graph representation was created for baseline and follow-up samples. The graph layout was fixed across samples and computed from phenotypic relationships. In this implementation vertex radii are proportional to their relative abundance, allowing for rapid visual assessment of population-level increases and decreases. Graph edge thickness and color encode inter-population similarity based on the optimal transport (Sinkhorn) distance between marker expression distributions.

**Results:**

This representation enabled rapid identification of populations undergoing substantial change, such as the CD8+/IFNɣ+ population, which decreased from 63% to 17% of CD8+ T cells following treatment. Population changes across all fluorescence parameters were encoded in the graph edit distance (GED), which captures changes in population abundance and phenotypic shifts in marker space.

**Discussion:**

Future implementations can combine this expert-guided approach with unbiased clustering algorithms to enhance scalability and cross-platform harmonization. In our recently initiated clinical trials, we will apply OT to identify key shifts in tumor, immune, and stromal cell states, summarizing patient trajectories and quantitatively supporting predictive models of treatment response. Potential applications include quantifying residual disease after chemotherapy, tracking immune activation during immunotherapy, and linking host–microbiome interactions to disease progression. This approach overcomes the limitations of traditional, local-structure-optimized tools (UMAP or t-SNE) to provide a comprehensive, longitudinal view of tumor evolution and treatment response.

## 1 Introduction

### 1.1 Visualization of intra-cavitary heterogeneity of malignant pleural effusions and peritoneal ascites

The pleural and peritoneal cavities are common sites for metastasis for epithelial cancers. Metastasis is often accompanied by accumulation of pleural or peritoneal fluids and carries a dire prognosis (1). Because there is currently no effective therapy, treatment consists of routine drainage to relieve symptoms of dyspnea and discomfort. Malignant pleural effusions (MPE) and malignant peritoneal ascites (MPA) are marked by striking heterogeneity (1–3), arising from a variety of primary tumors and shaped by an evolving and ultimately self-amplifying process in which tumor cells, immune cells and stromal cells interact to create a maladaptive and tumor promoting environment (1, 2). The availability of drained MPE or MPA, which are discarded as medical waste, has opened a window onto the biology of metastasis (1, 4–6), with ready access to tumor, infiltrating immune cells, as well as the cytokine-rich fluid that conditions the behavior of all populations.

Single-cell and spatial omics are capable of addressing MPE and MPA heterogeneity, revealing tumor cell states, T-cell functional capacity (7, 8) and myeloid polarization as they evolve during cytoreduction, regional therapies such as decortication and hyperthermic therapies (HIPEC), and systemic therapies (9–11). In this rapidly expanding data landscape, visualization tools, such as UMAP and t-SNE, that map high-dimensional data in 2-dimensional space, have become widely accepted tools for data exploration. Their utility has been demonstrated for visual hypothesis generation, quality control, and discerning patterns in complex datasets (12–14).

As useful as these visualization tools are for revealing hidden structures in high-dimensional data, inherent limitations preclude their use when the goal is to evaluate repeated sampling and longitudinal comparisons as is required when serially collected high-dimensional data sets are generated in clinical trials (15, 16). Here, we discuss a new alternative approach that focuses on capturing biological information as well as previously known domain information about the visualized data. This recently introduced methodology is manifold agnostic, which means it can use any notion of underlying similarity on the assumed manifold as input. Unlike earlier dimensionality reduction techniques, in which the visualization is directly driven by the supposedly recovered manifold, our implementation of optimal transport (OT) communicates biological notions of cellular heterogeneity, and how this heterogeneity is manifested in the phenotypic outcomes.

The approach utilizes the Sinkhorn algorithm, a computationally tractable, entropically regularized method that approximates earlier, less applicable OT methods. We assert that when integrated with contemporary machine learning (ML), these methodologies offer a systematic approach to the reliable, comparable quantification of clinically significant biological alterations (17–19). In the present work, we expand this perspective by combining OT-based quantification with a biologically interpretable, phenotype-aware graph framework that embeds expert-defined population relationships directly into the visualization. This framework draws on recently described methodology from Shemonti et al. (20), who demonstrated that the Sinkhorn distance provides a mathematically rigorous yet computationally tractable measure of inter-population similarity in high-parameter flow cytometry data. Building on this foundation, we pair these computational tools with an experimentally validated flow-cytometry panel and apply them to malignant pleural effusions and ascites collected before and after therapeutic intervention in an ongoing clinical trial.

Our aim is to illustrate how an OT-driven, domain-informed visualization approach can serve not only as an alternative to manifold learning, but as a reproducible and clinically meaningful method for summarizing immune and phenotypic structure in longitudinal samples. Specifically, this report (i) outlines conceptual limitations of manifold-learning visualizations for longitudinal clinical cytometry, (ii) describes an OT/Sinkhorn-based, phenotype-aware graph framework adapted from Shemonti et al. (20), and (iii) demonstrates its feasibility using high-parameter flow cytometry from malignant ascites collected during an ongoing intracavitary cytokine-blockade trial.

### 1.2 Limitations of UMAP and t-SNE in biological data analysis

Both UMAP and t-SNE methods are stochastic and, are sensitive to parameter configurations when applied in flow cytometry contexts. Empirically, they both excel in revealing local structure, inherent groupings and relationships (*e.g.*, immune cell phenotypic populations). In t-SNE, inter-cluster distances are meaningless. Although UMAP can preserve some global structure, it is more suited to finding local neighborhoods than global arrangements (16, 21). In t-SNE, parameters like perplexity and early exaggeration dictate the extent of neighborhood preservation (13). In UMAP, parameters such as the number of nearest neighbors and the minimum distance affect the density of local structures formed (12). Both methods are sensitive to batch effects and minor alterations in preprocessing, normalization, batch correction, or the incorporation of cells from a new time point, can yield markedly different outcomes (16, 22, 23).

In both t-SNE and UPMAP, graph axes and cluster size are without meaning, and cluster formations can shift or elongate, even if the underlying biology remains the same (16). Most importantly, comparison of data files requires that they be concatenated and analyzed together to achieve a consistent visual representation. For multiple comparisons across time and between subjects, this becomes computationally unfeasible.

As a result, while these methods remain useful for exploration and hypothesis generation, they are fundamentally unsuited to multi-subject longitudinal settings (15, 16). Clinical trials necessitate methods that can produce consistent results across multiple sites and time points while remaining dependable even when the underlying sampling varies (24). In this context, a visually consistent plot may be useful, but it does not always reflect a quantitative measure of true biological change (15, 16).

Flow and mass cytometry data present distinct challenges for UMAP because the measurement process itself contravenes the algorithm’s fundamental assumptions. In cytometry, fluorescent or metal-tagged antibodies attach to cellular markers, and detectors quantify photons or ions during short acquisition periods. The photon-counting process adheres to super-Poissonian distributions (such as the negative binomial) and is affected by technical variables including antibody affinity, excitation intensity, and detector sensitivity. The resultant data exhibit overdispersion, are frequently discrete, and display heteroskedasticity, rendering them not directly comparable, even locally, using Euclidean distances, thereby undermining the geometric assumptions foundational to UMAP.

Cytometry panels often include markers that differ in abundance by several orders of magnitude. Highly expressed surface proteins such as CD45 on leukocytes may yield hundreds of thousands of photon counts, whereas low-abundance transcription factors generate only tens of counts above background. When UMAP applies Euclidean distance to such data, high-intensity markers dominate distance calculations, rendering low-intensity markers nearly invisible. This distortion violates UMAP’s assumption of a locally constant Riemannian metric because the effective geometry of the space changes dramatically across markers. The binary search that UMAP uses to optimize local σ (sigma) values cannot compensate for this scale disparity.

The super-Poissonian distributions naturally create highly non-uniform densities in measurement space. Cells lacking expression for multiple markers cluster densely near the origin, whereas rare phenotypes occupy sparse peripheral regions. UMAP’s Lemma 1 assumes uniform sampling on the underlying manifold to justify its k-nearest-neighbor-based metric learning. When this assumption fails, σ values vary widely between dense and sparse regions, producing a “fuzzy simplicial set” with inconsistent geometric meaning. Neighborhood size, therefore, reflects sampling density rather than biological similarity.

These mathematical violations result in reproducibility failures. When the same cytometry dataset is processed with different random initializations or preprocessing pipelines, the resulting embeddings have radically different topologies. Rare populations can merge or vanish, and adjusting the number of nearest neighbors parameter does not smooth out resolution but instead causes abrupt cluster fragmentation or fusion. Technical artifacts, such as batch effects or compensation (demultiplexing) errors, can further distort the embedding because UMAP lacks a mechanism to distinguish measurement noise from biological variation when its geometric assumptions fail. The resulting visualizations may appear interpretable, but they have no stable mathematical relationship to the underlying biological structure.

### 1.3 Optimal transport as a framework to interpreting high-dimensional biological data

Data visualization through optimal transport (OT) offers an alternative method for encoding and visualizing high-dimensional biological data. Rather than attempting to “discover” the underlying manifold in order to reduce apparent dimensionality, it relies on domain knowledge and prior identification of specific functional cell populations. This identification can be accomplished through any technique, including supervised learning or even manual annotation. This method assumes that the data alone are insufficient, that they do not convey all of the necessary information about the biological context, and that prior domain knowledge, which is absent from the raw measurements, must also be incorporated into the visualization.

The visualization of heterogeneous phenotypes is thus achieved not by simply estimating the manifold and projecting the high-dimensional data to a two-dimensional plot regardless of domain knowledge, but by computing a notion of dissimilarity between the identified functional (and biologically meaningful and interpretable) cell populations. The concept of similarity is encoded in the amount of “work” required to transform one distribution of cellular states into another (25, 26). In omics applications, the Wasserstein distance, also known as the Earth Mover’s distance, captures the minimal cost of reallocating probability mass between samples such as pre- and post-treatment single-cell profiles (25). This calculation uses a biologically meaningful ground metric, which may be based on gene-expression distance, latent space distances from a variational autoencoder, or multimodal kernels.

The result is a single value that reflects the magnitude of change, where a larger transport cost indicates a greater shift at the population level (25, 26). Importantly, optimal transport works within the original or learned high-dimensional space rather than in a potentially unstable two-dimensional embedding, and it compares entire distributions instead of trying to match individual cells one by one (25, 27).

Shemonti et al. (20) describe the computational framework that underpins this perspective and introduce a graph-based visualization method based on optimal transport theory. In that study, cell populations are user-defined by marker-expression profiles, and inter-population similarity is measured using the Sinkhorn distance. These distances are embedded in phenotype-aware graphs, which enable both visual and quantitative comparisons between samples. The framework allows for robust tracking of biological changes in high-dimensional flow cytometry data, including longitudinal shifts in immune cell states during therapy, and has been validated in clinical and public datasets.

Our work builds directly on this computational foundation but does not introduce a new optimal-transport algorithm. Instead, we adapt the Sinkhorn-based framework of Shemonti et al. (20) to a new biological setting of malignant ascites in peritoneal carcinomatosis and integrate it with a rigorously validated flow-cytometry pipeline. This provides a practical demonstration of how OT-driven, phenotype-aware graphs can be incorporated into longitudinal clinical studies where reproducibility and interpretability are essential.

## 2 Methods

### 2.1 Patients and sample collection

Flow cytometry data were obtained from a single patient enrolled in the Regional Immuno-Oncology Trial 2 (RIOT-2), a Phase I study evaluating intraperitoneal delivery of the IL-6 receptor antagonist tocilizumab (NCT06016179) (3). The patient had malignant ascites due to metastatic appendiceal carcinoma and underwent therapeutic drainage via an indwelling catheter placed as part of standard care. Ascitic fluid was collected either at the pretreatment baseline or at a follow-up visit after initiation of therapy. All samples were assigned accession numbers and de-identified prior to analysis.

Ascites were drained aseptically into 1000 mL vacuum bottles (Becton Dickinson, SKU/REF 50-7700). Upon receipt at the Allegheny Health Network Cellular Therapy Laboratory, preservative-free sodium heparin was added to a final concentration of 10 U/mL. After aliquots were removed for bacterial and fungal cultures, penicillin G (40 µg/mL) and gentamicin (100 U/mL) were added. Samples were filtered through a 170–260 μm filter (Baxter, 2C8750 Blood/Solution Set), and a de-identified aliquot was transferred to the UPMC Hillman Cancer Center research laboratory for downstream flow cytometry (MTA00011955). This clinical setting is an ideal test case for OT-based visualization, as repeated sampling of the peritoneal compartment is feasible, yet traditional manifold-learning approaches fail to provide stable, interpretable longitudinal comparisons.

### 2.2 Institutional review board protocol and informed consent

This study was conducted in accordance with the Declaration of Helsinki and approved by the Allegheny Health Network Cancer Institute Institutional Review Board for the Regional Immuno-Oncology Trial 2 (RIOT-2), a Phase I study of intrapleural and intraperitoneal tocilizumab (IRB RC# 2023-137; NCT06016179). Flow cytometry analyses in this report were performed on de-identified malignant ascites specimens collected under this approved protocol. All samples were anonymized prior to research use in accordance with institutional guidelines.

Written informed consent was obtained from the patient prior to enrollment in the RIOT-2 clinical trial and prior to collection of malignant ascites for research purposes, including exploratory flow-cytometric and computational analyses. All samples used in this study were de-identified before analysis in accordance with the approved protocol.

### 2.3 Data availability statement

Data will be made available to qualified investigators upon request to the corresponding author.

### 2.4 Flow cytometry for surface marker expression

Sample preparation and staining for surface markers followed protocols previously established by Donnenberg and colleagues for analysis of immune subsets and tumor-associated stromal populations (28, 29). Malignant ascites cells were washed in staining buffer (4% calf serum, 2 mM EDTA in Ca^2+^/Mg^2+^-free PBS, pH 7.2, 4 °C). Cell pellets were incubated with neat decomplemented mouse serum (10 µL, 5 min, 4 °C) to block Fc-mediated nonspecific binding, followed by staining with 2 µL each of fluorescently conjugated antibodies targeting surface markers. A complete list of antibodies, fluorochromes, catalog numbers, and vendors appears in **Table 1**.

**Table 1:** Antibodies used for flow cytometry of surface proteins and intracellular cytokines.

Analyte	Fluorochrome	Catalog #	Vendor
CD3	BV711	563725	BD Horizon
CD4	BV785	317442	BioLegend
CD8	ECD	660478	Beckman Coulter
CD14	PerCP Cy5.5	325622	BioLegend
CD45	BUV395	563792	BD Horizon
CD56	PE Cy5	IM2654U	Beckman Coulter
CD19	APC-A700	A78837	Beckman Coulter
CD326	APC Cy7	324234	BioLegend
DNA	DAPI	D1306	Invitrogen
TNFα	FITC	502915	BioLegend
IL-10	PE	506804	BioLegend
lFNγ	PECy7	506518	BioLegend
IL-2	APC	500310	BioLegend

CD, cluster of differentiation; BV711 and BV785, Brilliant Violet 711 and Brilliant Violet 785; BD, Becton Dickinson; ECD, phycoerythrin–Texas Red tandem fluorochrome; PerCP-Cy5.5, peridinin–chlorophyll-protein–Cyanine5.5 tandem fluorochrome; BUV395, Brilliant Ultraviolet 395; PE-Cy5, phycoerythrin–Cyanine5 tandem fluorochrome; APC-A700, allophycocyanin–Alexa Fluor 700 tandem fluorochrome; APC-Cy7, allophycocyanin–Cyanine7 tandem fluorochrome; DNA, deoxyribonucleic acid; DAPI, 4′,6-diamidino-2-phenylindole; TNF, tumor necrosis factor; FITC, fluorescein isothiocyanate; IL, interleukin; PE, phycoerythrin; IFNγ, interferon gamma; PECy7, phycoerythrin–Cyanine7 tandem fluorochrome; APC, allophycocyanin.

After staining, cells were fixed in 2% methanol-free formaldehyde (Polysciences, Cat. No. 0401A) for 20 minutes, permeabilized with 0.05% saponin (Coulter) in staining buffer, washed, and resuspended to 5 × 10^6^ cells/mL. Prior to acquisition, DAPI (Invitrogen D1306, 10 µg/mL) was added for DNA content assessment and identification of cycling CD3^+^ cells.

All samples were acquired on a Fortessa SORP flow cytometer (BD Biosciences, Hillman Cancer Center Cytometry Core). Daily instrument calibration was performed using CS&T beads (BD Biosciences, Cat. No. 650621). PMT voltages were adjusted to predetermined target channels using the seventh peak of 8-peak Rainbow Calibration Particles (Spherotech RCP-30-5A). Spectral compensation was derived from FITC, PE (BD 349502), and APC (BD 340487) Calibrite beads, single-stained BD CompBeads (anti-mouse Igκ; BD 51-90-9001229) for tandem dyes, and unstained DAPI-only cells.

### 2.5 Flow cytometry for intracellular cytokine profiling

Intracellular staining for IFNγ, TNFα, IL-10, and IL-2 followed methods previously described for cytokine profiling in pleural infiltrating T cells (1). Cells were washed and resuspended in complete medium (RPMI-1640 supplemented with 10% FBS) at 2 × 10^6^ cells/mL. A 4 mL suspension was plated in 15 mL polypropylene tubes and incubated for 1 hour at 37 °C with tetradecanoylphorbol-13-acetate (TPA, 0.05 µM) and ionomycin (1 µM) (30). Cells were then washed, resuspended in complete medium, and treated with Brefeldin A (1 µg/mL) for two additional hours at 37 °C to inhibit cytokine secretion.

Following stimulation, cells were washed and blocked with decomplemented mouse serum (10 µL, 5 min, 4 °C), stained for surface markers as above, fixed with 2% methanol-free formaldehyde, permeabilized with 0.05% saponin, and washed. Cells were split into two equal aliquots, one of which was stained with cytokine-specific antibodies (listed in **Table 1**). Samples were then treated with PureLink RNase A (Invitrogen, 1:400, 10 min, 37 °C), washed, resuspended to 5 × 10^6^ cells/mL, and stained with DAPI prior to acquisition on the Fortessa SORP cytometer.

**Figure 1:**
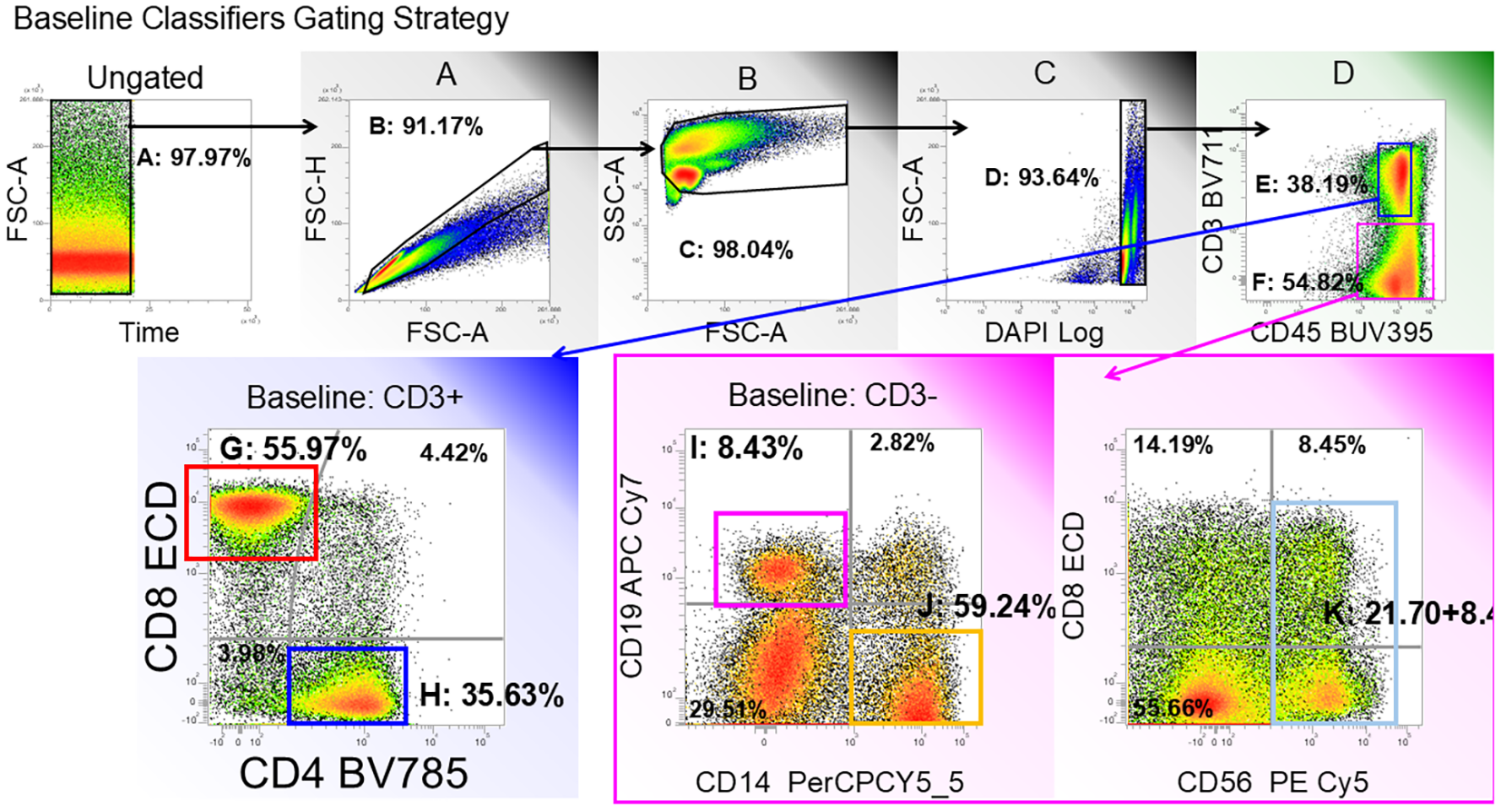
Manual Gating Strategy. From left to right, top to bottom: Forward light scatter (FSC) area versus time is used to detect fluidics perturbations that might result in artifact (here none were detected). **(A)** FSC pulse area versus FSC pulse height is used to identify and gate out events with pulse area too great for the pulse height (Outside gate B, doublets and multiples and small debris). **(B)** FSC area versus side light scatter (SSC) area is used to gate our small debris (Outside gate C). **(C)** DAPI versus FSC is used to gate out events with less then 2n DNA (note that both 2n and 4n populations are visible within the D gate). **(D)** CD45 and CD3 are used to distinguish between T cells (E gate) and non-T leukocytes (F gate). T cells are further gated on CD8+ T cells (G gate) and CD4+ T cells (H gate). CD14 vs CD19 is used to identify B cells (I gate), macrophages (J gate) and NK cells (K gate) among non-T leukocytes. Cytokines were measured as “outcomes” on these “classifier” populations. This gating strategy was used for all baseline and follow-up samples.

Cytokine gating thresholds were defined using samples stained only for extracellular markers (no intracellular cytokines). Data analysis was performed using VenturiOne software (Applied Cytometry Systems, V7.6.0.47.X64). DAPI staining enabled estimation of the proportion of CD3^+^ cells in cycle (DNA >2N). The full gating strategy is shown in **Figure 1**.

### 2.6 Antibody reagents

All antibody reagents are summarized in **Table 1**, including marker, fluorochrome, catalog number, and vendor. Surface markers included lineage and functional proteins (CD3, CD4, CD8, CD14, CD19, CD45, CD56, CD326), and intracellular cytokine antibodies targeted IFNγ, TNFα, IL-10, and IL-2. DAPI was used for DNA content.

### 2.7 Data preprocessing and cell population identification

Computational preprocessing drew on established approaches for flow cytometry data annotation and semi-automated gating, consistent with previously published pipelines. Data were imported from FCS files and quality-controlled by removing debris and margin events. Surface and intracellular markers were compensated and transformed as described above.

Population identification relied on a semi-automated, knowledge-driven gating schema derived from the explicit fluorescence-intensity boundaries defined during manual gating of representative samples. This approach parallels the strategy used in Shemonti et al. (31), where population labels, whether manually gated, heuristically defined, or produced by automated methods, serve as the required input for downstream optimal transport computations. Each cell was assigned to a biologically interpretable phenotype defined by marker expression (e.g., CD3^+^CD4^+^ T cells, CD3^+^CD8^+^ T cells, NK cells, CD14^+^ monocytes). These population definitions form the categorical structure on which optimal transport distances are computed.

As described by Shemonti et al. (31), this framework is fully agnostic to the upstream labeling method: the core requirement is that each cell belongs to a defined population. This preserves interpretability and ensures compatibility with domain knowledge and prior cytometric expertise.

### 2.8 Optimal transport computation and graph-based visualization

Inter-population dissimilarity was quantified using the entropically regularized optimal transport (OT) framework described by Shemonti et al. (20) and based on the Sinkhorn distance. A plain-language description of the OT approach to high-dimensional flow cytometry data is provided in **Supplementary Materials**: Optimal Transport Basics.

For any two populations *C*_1_ and *C*_2_, each cell was treated as a unit mass normalized to total population size (1/n_1_ and 1/n_2_). The ground cost matrix was computed as the squared Euclidean distance between marker-expression vectors, consistent with the formulation presented in the preprint.

The Sinkhorn–Knopp algorithm was used to compute regularized transport plans, enabling efficient scaling to large cell counts. The resulting Sinkhorn distances reflect high-dimensional distributional differences between cell populations and form the quantitative backbone of the visualization framework.

To construct interpretable sample-level visualizations, we adopted the graph-based approach described in Shemonti et al. (20). Each cell population corresponds to a vertex, with vertex size proportional to its frequency within the sample. Edges encode inter-population Sinkhorn distances, visualized through thickness and color intensity. Vertex positions were determined by fixed, phenotype-aware layouts using Hamming distances between expert-defined phenotype strings, ensuring reproducible and biologically meaningful geometry.

This fixed layout allows multiple samples to be compared directly, and changes in edge weights reflect shifts in marker-expression distributions across populations. As in the preprint, these graphs serve as compact, interpretable “fingerprints” of sample structure. Graph edit distance (GED), with biologically informed vertex and edge substitution costs, can be used to quantify dissimilarity between samples and is well-suited for longitudinal comparisons in future multi-timepoint analyses.

### 2.9 The Sinkhorn algorithm and machine learning integration

While our present application focuses on a single-patient proof-of-concept, the same computational framework scales to multi-patient cohorts and supports machine-learning workflows designed for longitudinal PC trials. The Sinkhorn algorithm makes optimal transport feasible at the scale required for processing large datasets, such as those generated in clinical trials, where serial observations are made to track responses (19, 32, 33). By adding entropic regularization, the classical transport problem becomes a convex, well-conditioned objective that can be solved efficiently via parallel matrix scaling (19, 33). This approach performs well on GPUs and scales to very large datasets with appropriate batching (19, 32).

Sinkhorn divergences also help correct for bias introduced by regularization, which allows meaningful comparisons even when sample sizes are unequal, as is often the case in cells taken from serial tumor or ascites sampling (17, 18). In addition, optimal transport barycenters can summarize groups of patients or define reference atlases of peritoneal carcinomatosis microenvironments, such as immune-active, myeloid-dominant, or mesothelial-fibrotic states (27, 34). Each patient’s samples can then be measured against these references, turning endpoints into shifts in transport distance rather than movements of clusters in a two-dimensional plot (25, 27).

Machine learning fits naturally into this framework. Deep generative models such as variational autoencoders provide denoised, batch-corrected latent spaces that preserve biological structure (35). Optimal transport then supplies geometry-aware distances, alignments, and trajectories within these spaces (36). This view also supports domain adaptation, where transport minimizes differences between sites or platforms, such as CyTOF, single-cell RNA sequencing, and spatial proteomics, thereby improving comparability across centers in multicenter trials (27, 36). Transport plans can identify which cell states change most after therapy, highlighting, for example, shifts in proliferative EpCAM-positive tumor cells, interferon-responsive macrophages (37, 38), CXCL13-positive Tfh-like cells, or mesothelial cells with high mesothelin expression (9, 25).

These features can then be used as inputs to predictive models of treatment response, progression-free survival, or the transition from an immune-cold to an immune-hot microenvironment (39). This approach allows machine learning models to learn from distributions of cell states rather than from potentially unstable two-dimensional representations (35).

## 3 Results

### 3.1 Applications for sequential drainage of malignant pleural effusions and ascites: an example from peritoneal carcinomatosis MPA

In peritoneal carcinomatosis trials, optimal transport and machine learning can be applied directly to clinically meaningful endpoints (31, 40, 41). In the context of immunotherapy or cytokine-targeted treatments, optimal transport can define mechanistic distributional shifts (42–44), such as movement from a patient’s baseline toward an atlas enriched for activated T cells, transitions into or out of macrophage programs linked to immunosuppression (45), or redistribution among epithelial EMT states that drive peritoneal spread (39, 46–48). For cytoreduction, intrapleural and hyperthermic intraperitoneal chemotherapy studies, transport distances can quantify how residual disease evolves after surgery, capturing shifts toward quiescent or stress-response phenotypes (9, 49, 50).

When the peritoneal microbiome is incorporated, multimodal transport, or machine learning co-embedding followed by transport, can identify coordinated changes between microbial communities in ascites and host immune states such as myeloid polarization or cytokine signaling (27). Unlike two-dimensional embeddings, these measurements can be applied more consistently across time points and trial centers, providing a stable and reproducible way to evaluate biological change (25, 27). These analyses can quantify distributional shifts, but links to clinical response require prospective validation (25, 51). Together, these examples illustrate how transport-based approaches provide a practical and reproducible framework for clinical research in PC (25, 27, 36). Although these applications extend beyond the scope of the present demonstration, they outline realistic future directions for integrating OT-based cytometry endpoints into clinical trials.

**Figure 2:**
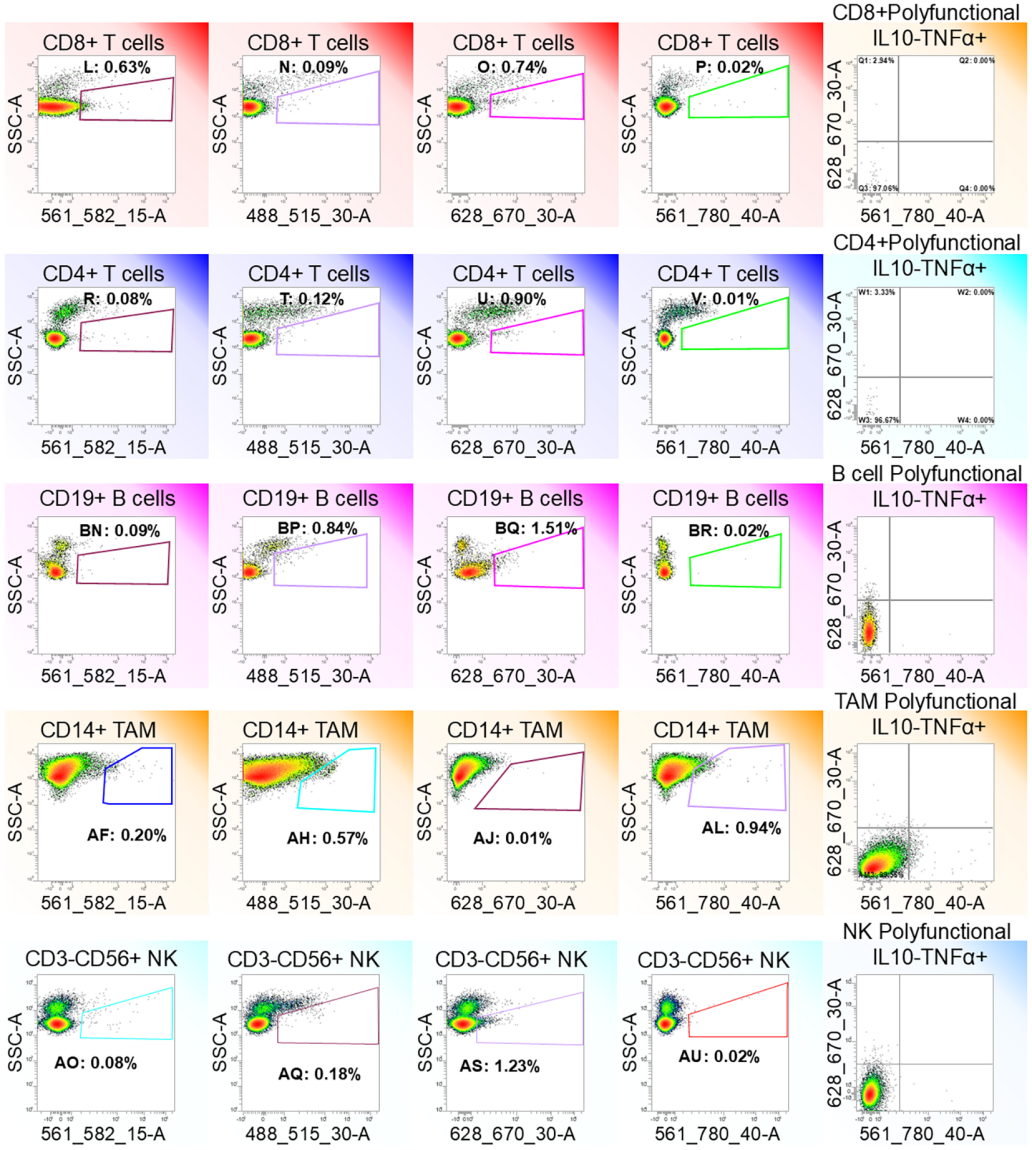
Baseline fluorescence minus outcomes plots. A separate tube, stained for the “classifier” populations, but not cytokine “outcomes”, was used to determine the boundaries between cytokine positive and negative populations. CD4+ T cells are shown in the top histograms (red border.) and CD8+ T cells are shown in the bottom histograms (blue borders). Note autofluorescence or non-specific staining in the high SSC population. The same strategy was used for B-cell, macrophage and NK-cell classifier populations.

In this demonstration, we have applied t-SNE, UMAP and OT to malignant ascites samples collected before and after an immunotherapeutic intervention. Cells recovered from the ascitic fluid were stimulated in short-term culture with TPA plus ionomycin (TPA+I) and stained to measure intracellular cytokines in different immune cell populations. **Figure 1** (Baseline gating strategy) shows the gating strategy used for conventional flow cytometric analysis, creating *classifier* populations (52) (CD4+ and CD8+ T cells, B cells, macrophages, NK cells) in which cytokine *outcomes* (IL-2, IL-10, interferon-γ, and TNF-α) are measured. **Figure 2** is gated on the 5 *classifier* populations and shows the fluorescence intensity of the channels that are used to measure the four cytokines (fluorochromes minus outcomes controls (52)).

**Figure 3:**
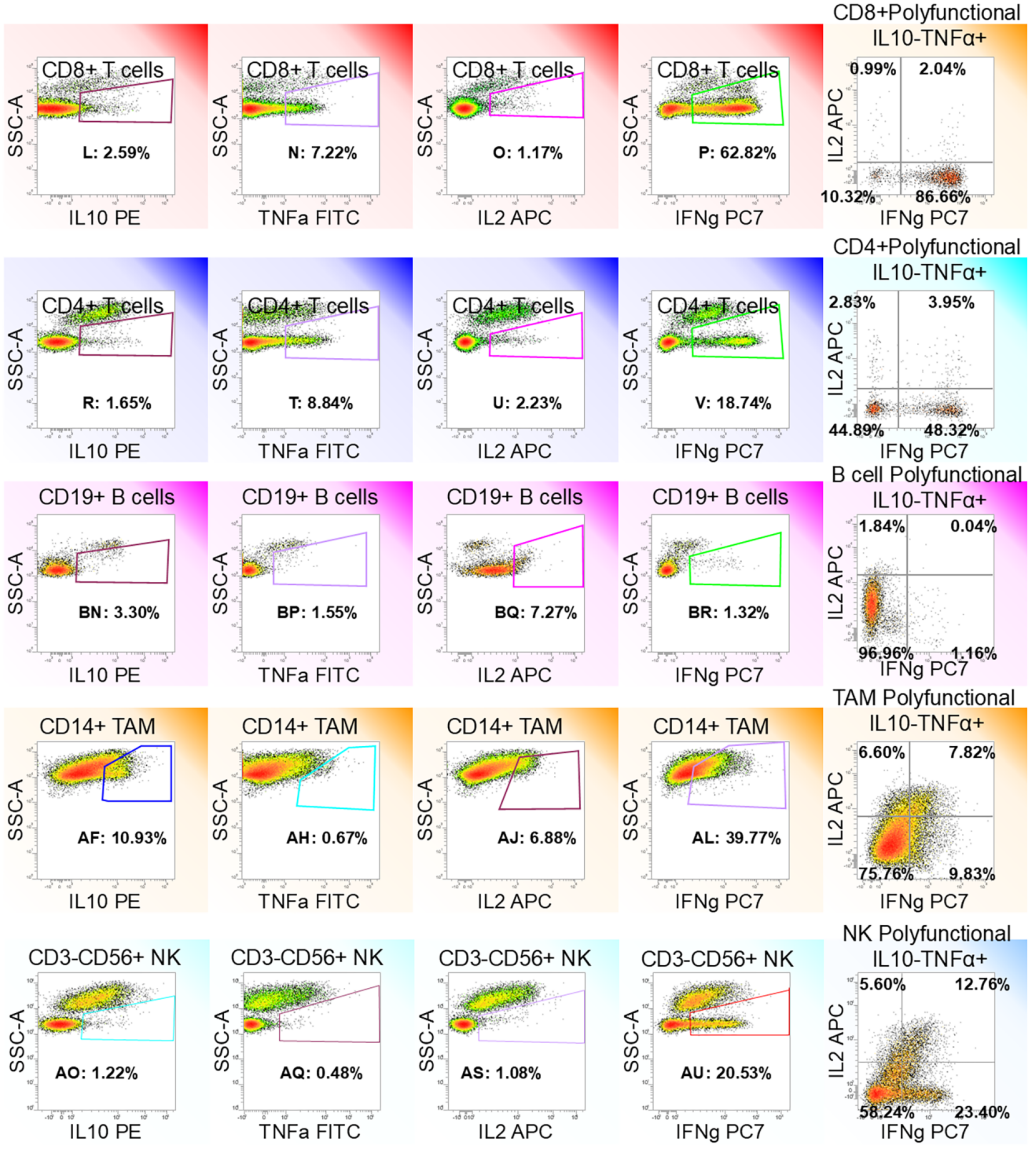
Baseline cytokine outcomes. Histograms are in the same order as the fluorescence minus outcomes control. Rows from top to bottom: CD4+ T cells (red border); bottom row: CD8+ T cells (blue border), B cells (pink border), macrophages (gold border), NK cells (light blue border). Histograms to the far right are gated on the IL-10 negative, TNF-alpha positive population and are used to determine polyfunctionality. Note that significant populations of CD4+ and CD8+ IL-10-/TNF+ T cells also secrete IFN-gamma.

**Figure 4:**
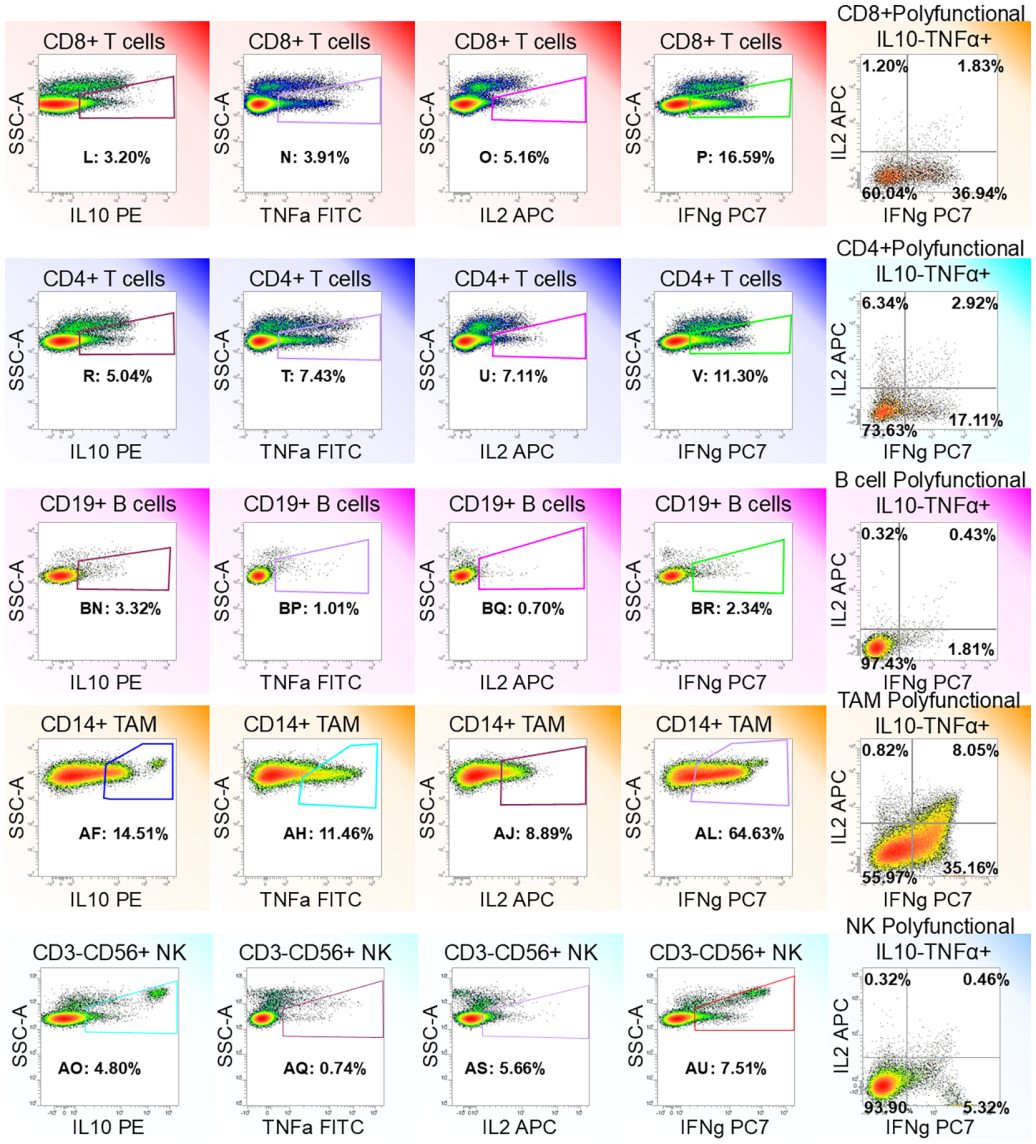
Follow-up cytokine staining. Histograms are arranged in the same order as for baseline cytokine staining. IFN-gamma secretion was markedly down-regulated and IL-2 secretion is increased in CD4+ and CD8+ populations compared to baseline, while TNF-alpha and IFN-gamma secretion increased in macrophages.

The regions were drawn to include ≤ 1% of events and were applied to the data files stained for both *classifier* and *outcome* analytes (**Figures 3**, **4**). **Figure 3** shows intracellular cytokines in the ascites sample collected at baseline. At baseline, TPA+I stimulation elicited modest TNF-α responses and robust IFN-γ responses in CD4+ and CD8+ T cells. Macrophages and NK cells also produced IFN-γ. Following tocilizumab treatment IL-2 appeared to be upregulated in CD4+ and CD8+ T cells, TNFα was upregulated in macrophages, and IFN was downregulated in T- and NK cells but upregulated in macrophages (**Figure 4**).

**Figure 5:**
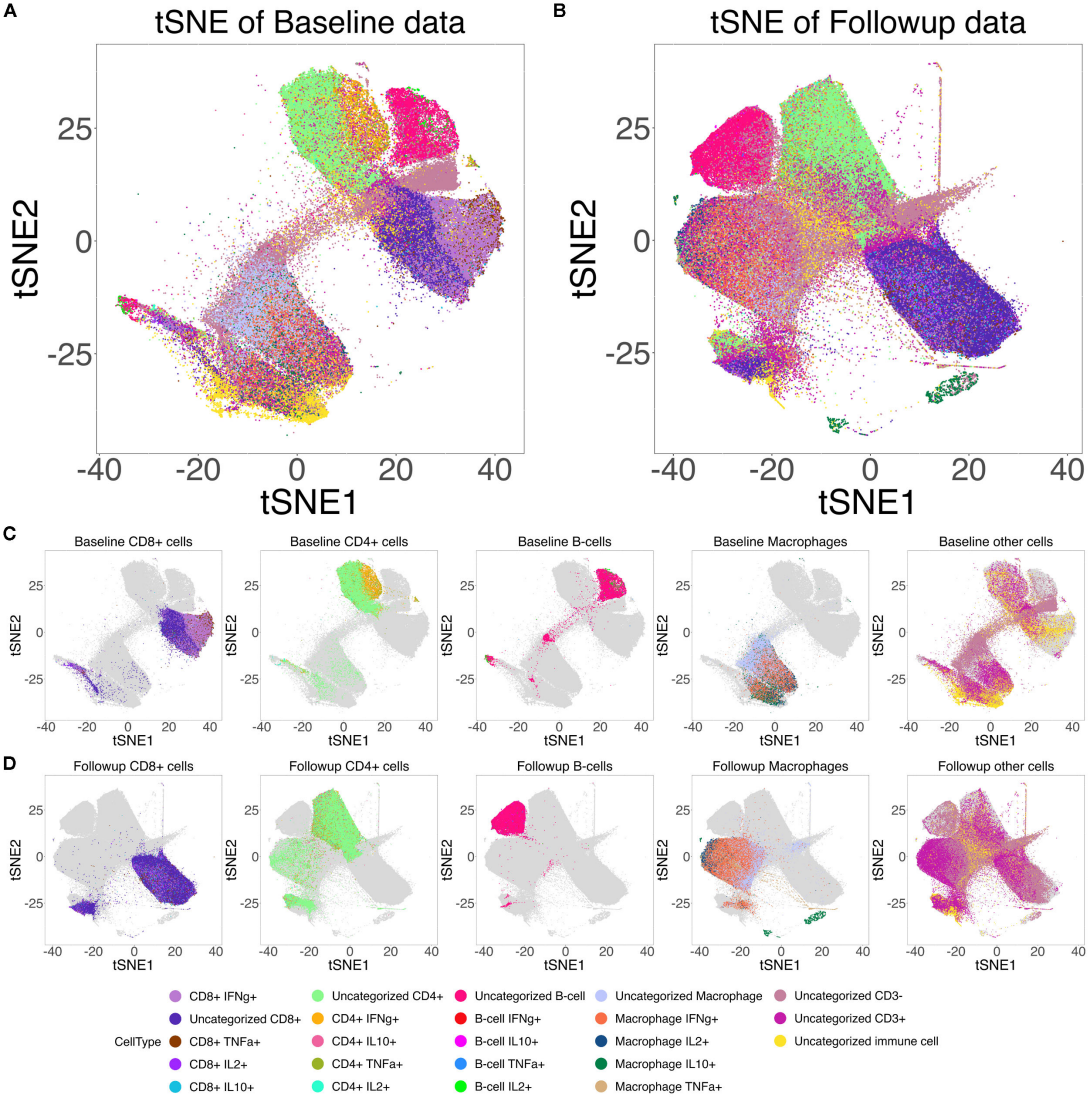
T-SNE visualization of baseline and follow-up flow cytometry samples. T-SNE embeddings of baseline and follow-up flow cytometry datasets are shown. A shared low-dimensional reference embedding was first computed from a combined, cell population density-aware subsample of both datasets to ensure representation of all annotated cell populations while enabling scalable computation. All cells from the full baseline and follow-up datasets were then projected into this common T-SNE space. T-SNE was generated in R (version 4.4.1) using the Rtsne package on macOS Sequoia (version 15.4.1), with a perplexity of 7. **(A)** T-SNE embedding of the full baseline dataset, with all cells projected into the shared reference space. **(B)** T-SNE embedding of the full follow-up dataset, projected into the same reference space. **(C)** Baseline T-SNE embeddings restricted to selected major immune populations (CD8+ T cells, CD4+ T cells, B cells, macrophages, and uncategorized cells), shown separately to highlight population-specific structure within the baseline sample. **(D)** Follow-up T-SNE embeddings of the same selected immune populations (CD8+ T cells, CD4+ T cells, B cells, macrophages, and uncategorized cells), as in **(C)**, facilitating qualitative comparison of population distributions between baseline and follow-up conditions.

**Figure 5** shows the data from **Figures 3**, **4**, projected as t-SNE plots. Data were gated on CD45+ events. Events are colored according to their analyte expression by conventional flow cytometric analysis. The plots are dominated by uncategorized (*i.e*., cytokine negative) CD8+ (purple), CD4+ (green) T cells, and B cells (hot pink). Positional and size changes of these clusters after therapy are without meaning. Most cytokine+ populations are inapparent, with the exception of CD8+/IFN-γ+ cells (light purple), which comprised 63% and 17% of CD8+ T cells at baseline and follow-up, respectively. This decrement in IFN-γ+ T cells after therapy was not captured in the t-SNE plot, because cluster area does not represent population prevalence.

**Figure 6:**
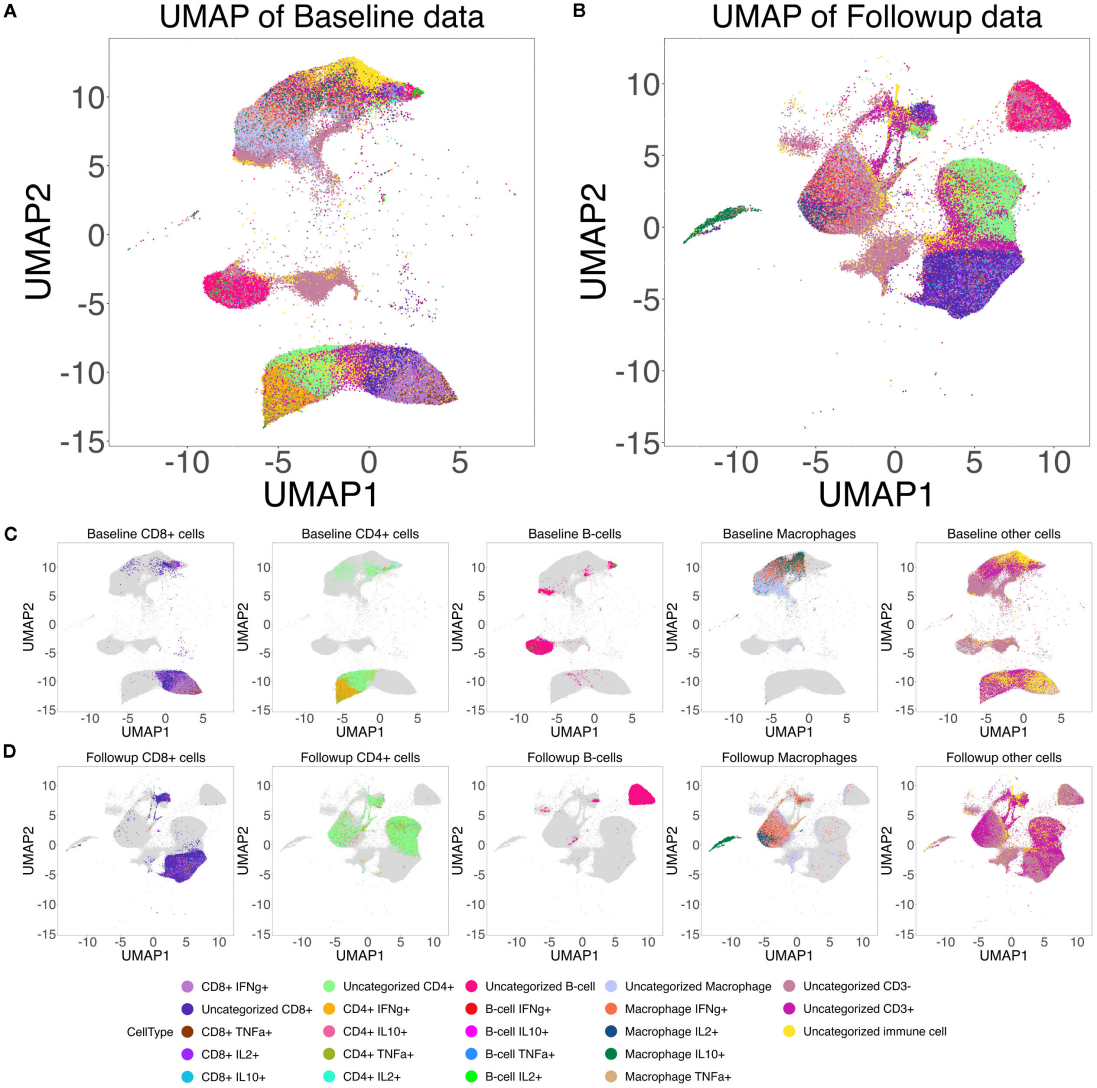
UMAP visualization of baseline and follow-up flow cytometry samples. UMAP embeddings of baseline and follow-up flow cytometry datasets are shown using a shared reference-based embedding strategy (**Figures 3**, **4**). A combined, cell population density-aware subsample from both datasets was used to compute a joint UMAP reference, preserving representation of all annotated cell populations while reducing computational complexity. The complete baseline and follow-up datasets were subsequently projected into this shared UMAP space. UMAP was computed in R (version 4.4.1) using the uwot package on macOS Sequoia (version 15.4.1), with n_neighbors = 15. **(A)** UMAP embedding of the full baseline dataset, with all cells projected into the shared reference space. **(B)** UMAP embedding of the full follow-up dataset, projected into the same reference space. **(C)** Baseline UMAP embeddings restricted to selected immune populations (CD8+ T cells, CD4+ T cells, B cells, macrophages, and uncategorized cells), shown separately to emphasize population-specific organization within the baseline sample. **(D)** Follow-up UMAP embeddings of the same immune populations (CD8+ T cells, CD4+ T cells, B cells, macrophages, and uncategorized cells), as in **(C)**, enabling visual assessment of changes in population structure and distribution between baseline and follow-up samples.

**Figure 6** shows the same data in UMAP plots. As in the t-TNE plots, the graphs are dominated by CD4+, CD8+ T cells and B cells, with the minor cytokine+ populations obscured. However, homologous populations from the two samples are greatly displaced in 2D space (e.g., the B-cell clusters, shown in hot pink). This artifact is due to UMAP’s susceptibility to subtle batch effects. Within samples, some global structure is preserved, as CD4+/IFN-γ+ cells (gold) are largely clustered within CD4+ cells, and CD8+/IFN-γ+ cells (light purple) are clustered within CD8+ cells. Overall, these plots entirely fail to capture quantitative therapy-related changes in cytokine production and are insensitive to minor cytokine+ populations.

**Figure 7:**
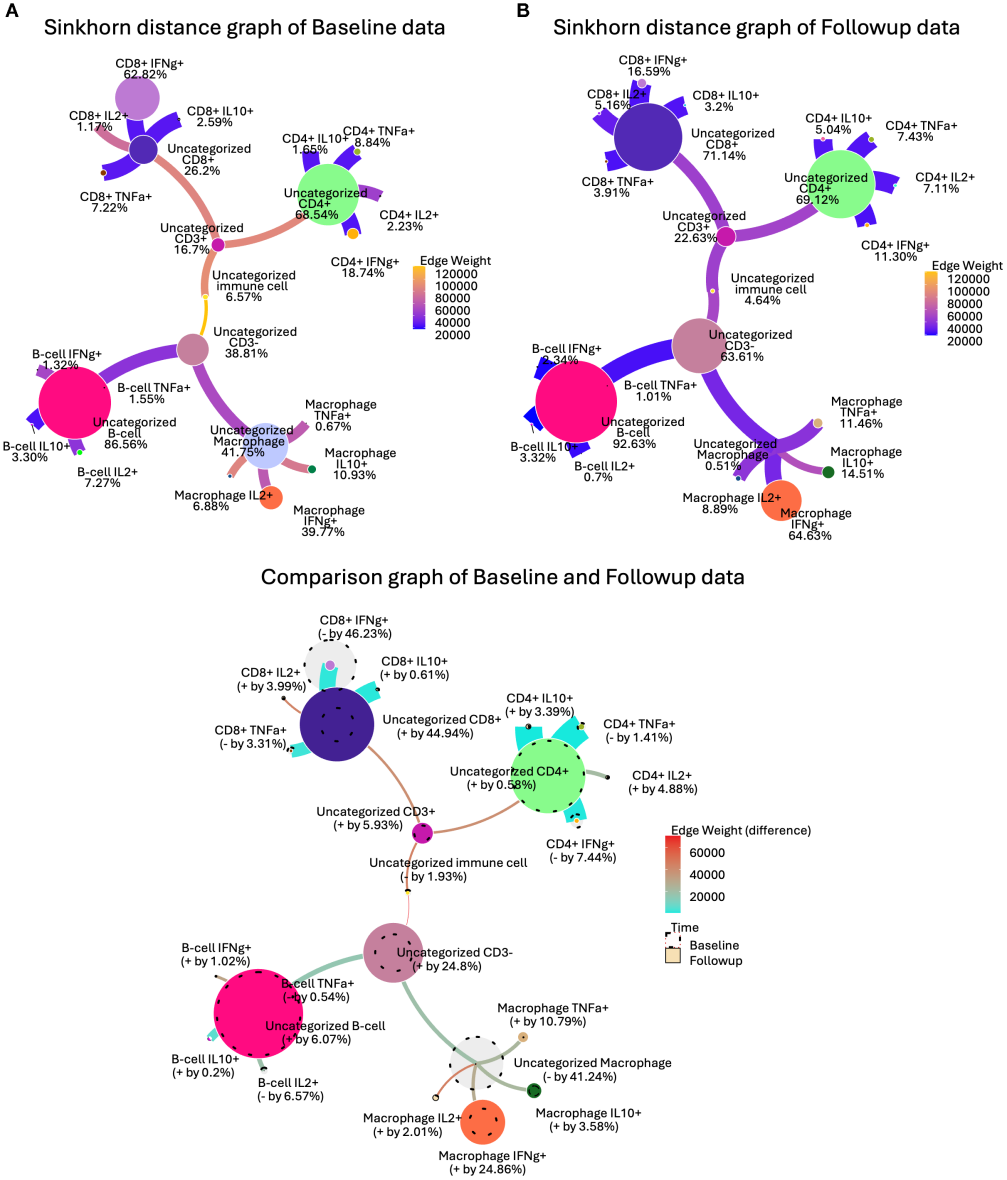
OT analysis visualization of data provided in **Figures 3**, **4**. Sinkhorn distance-based visual summary of changes in classifier populations and outcomes before (baseline) and after (follow-up) intraperitoneal administration of tocilizumab. Illustrations of the flow cytometry sample graphs at baseline **(A)** and one week after completion of 4 therapeutic doses **(B)**. The vertices (circles) represent individual classifier populations identified manual gating. Their radius is proportional to their relative proportion among total valid events (CD45+ positive events identified by the gating strategy). Vertex positions are determined by pairwise phenotype distances, so phenotypically similar populations lie closer together. Edges connect the vertices. Their weights (but not lengths), indicated by color and line thickness, encode the inter-population Sinkhorn distances: smaller distances indicating greater similarity in marker expression produce thicker, more vividly colored edges, while larger distances yield thinner, paler edges. Bottom panel: Vertices from the two samples are distinguished by their outlines: The baseline sample’s vertices appear as dotted circles and the follow-up’s as solid circles. When a population’s proportion increases, the solid circle encloses the dotted one; when it decreases, the dotted circle is larger. Numeric labels at each vertex indicate the percentage change in that cell population. Edges encode the absolute change in Sinkhorn distance between the two samples. A positive edge weight denotes increased similarity (i.e., a decrease in Sinkhorn distance), whereas a negative weight denotes decreased similarity (i.e., an increase in Sinkhorn distance).

**Figure 7** illustrates the optimal transport–based graph representation of the same baseline (**Figure 3**) and follow-up (**Figure 4**) samples. Our implementation of OT analysis begins with manual gating, as in **Figures 1**–**3**. The graph layout is fixed across samples and computed from phenotypic relationships among cell populations, ensuring positional consistency and enabling direct visual comparison between time points. Vertices represent cell populations, with vertex radii proportional to their relative abundance with respect to the immediate parent population defined during manual gating. This encoding allows rapid visual assessment of population-level increases and decreases between baseline and follow-up samples. In addition, the shared layout enables direct graph overlay: in the third panel of **Figure 7**, baseline populations are shown with dotted outlines and follow-up populations with solid outlines, facilitating intuitive identification of treatment-associated changes.

Edge thickness and color encode inter-population similarity based on the optimal transport (Sinkhorn) distance between marker expression distributions. Thicker and more saturated edges indicate greater phenotypic similarity, whereas thinner and paler edges correspond to increased dissimilarity. In the overlay graph, edges represent differences in Sinkhorn distance between baseline and follow-up samples, highlighting population pairs exhibiting the most pronounced phenotypic shifts.

This representation enables rapid identification of populations undergoing substantial change, such as the CD8+/IFN-γ+ population (light purple), which decreased from 63% to 17% of CD8+ T cells following treatment. Beyond visualization, encoding flow cytometry samples as optimal transport–based graphs allows quantitative comparison using graph-theoretic metrics. In particular, graph edit distance (GED) captures both changes in population abundance (via vertex size) and phenotypic shifts in marker space (via Sinkhorn distances). While individual GED values are not directly interpretable in isolation, pairwise GEDs across a patient cohort enable downstream statistical analyses, such as pseudo-ANOVA, to assess treatment effects and compare therapeutic strategies.

## 4 Discussion

UMAP and t-SNE remain useful tools for data visualization and exploratory data analysis. They can be used to perform rapid dimensionality reduction, which is helpful in the identification of unique populations that can only be visualized in multidimensional space, for revealing integration artifacts, and generating biologically grounded hypotheses. In multiparameter flow cytometry, such the data set provided here, they are useful for exploratory analysis of the tumor microenvironment, immune responses, and host–microbiome interactions (12, 13). However, employing these methods as a preprocessing step prior to unsupervised clustering is methodologically problematic. UMAP’s theoretical framework assumes data is locally uniformly distributed on the underlying manifold (12), which is fundamentally incompatible with biological single-cell data where cells form discrete, locally clustered populations. When this assumption is violated (as it inherently is with clustered cell types) UMAP introduces substantial distortions: it fails to preserve majority of nearest neighbors, distorts global cell-type relationships (correlation ≤0.4 with high-dimensional space), and inflates distance ratios 4–200 fold (53). These distortions make the 2D coordinates unreliable for clustering algorithms that depend on accurate distance metrics. For these reasons, it has been argued that clustering should be performed in the original high-dimensional space or in a dimensionality-reduced space that preserves global structure (e.g., PCA), with UMAP/t-SNE used solely for visualization of the resulting clusters (12).

As a result, while manifold learning’s limitations do not affect data exploration, they can limit their usefulness for longitudinal clinical studies in the flow cytometry field, where the objective is to quantify biologically meaningful changes occurring over time (16, 31, 54). In contrast, optimal transport-based analysis and visualization, which are efficiently implemented with the Sinkhorn algorithm and combined with machine learning, provide the stability and comparability that trials require (27, 36). As cancer research becomes more multi-omic and multicenter, with longitudinal follow-up, transport-based endpoints can transform the complexity of single-cell and spatial data into measures that are clinically interpretable (25, 27, 36). This approach emphasizes the underlying biological changes represented by data visualizations rather than their appearance (25, 27).

In this manuscript, we provide a proof-of-concept example demonstrating how integrating OT with phenotype-aware graph construction yields a reproducible, biologically interpretable representation of flow-cytometry samples collected from a patient undergoing intraperitoneal cytokine blockade (1, 2). This approach is directly informed by the methodological advances described by Shemonti et al. (20), particularly the use of the Sinkhorn distance as a robust statistical measure of inter-population similarity. By anchoring the visualization in explicit phenotype definitions rather than stochastic manifold embeddings, we recover a structure that faithfully reflects both proportional shifts in cell populations and distributional changes in marker expression.

Because the framework accommodates both longitudinal sampling and heterogeneous clinical datasets, it provides a foundation for trial-ready cytometry endpoints capable of capturing immune modulation, treatment response, and evolving tumor–immune interactions in PC. As OT-based methodologies continue to mature, they offer a path toward unified, reproducible, and quantitatively interpretable visualization standards for high-parameter cytometry in clinical research.

In this proof-of-concept analysis, we demonstrate that optimal-transport–based, phenotype-aware graph representations can reliably summarize high-parameter flow-cytometry profiles from malignant effusions and ascites and reveal biologically interpretable shifts that are obscured in conventional manifold-learning visualizations. We are currently applying this methodology to our longitudinal clinical trial data in malignant effusions and ascites (1, 2) and anticipate further refinements facilitated by integration with existing flow cytometry packages. The small dataset presented here represents one panel among four currently in use to monitor two Phase I clinical trials of an adoptive cellular therapeutic (RIOT4A, NCT07192900, RIOT4B, NCT07443020), the others being T-cell activation, immune checkpoint expression, and T-cell differentiation. Future applications include expanding the OT framework to these panels in the RIOT2 patient cohorts, comparing it to conventional analysis, and evaluating the extent to which OT-based endpoints track therapeutic response.

## Data Availability

The original contributions presented in the study are included in the article/**Supplementary Material**. Further inquiries can be directed to the corresponding author.
