# Supplementary material for: Optimal transport analysis of high-dimensional flow cytometry data in immuno-oncology
Source: Front Immunol. 2026 Jul 28;17:1856896. doi: 10.3389/fimmu.2026.1856896 (PMC13457739; doi:10.3389/fimmu.2026.1856896)
Supplement: Supplementary file 1 [file DataSheet1.pdf]

Application of the Optimal Transport (OT) framework to Flow Cytometry Data

The purpose of this supplement is to explain our application of OT in plain language.

The Goals

- To visualize flow cytometry data in low-dimensional space incorporating what is known about the individual cell phenotypes of interest and the biological relationships of those cell phenotypes.
- To create a fixed layout for each population, related to that biology (a phenotype-aware layout).
- To measure changes in population prevalence and interpopulation relationships over time or with treatment, between individual samples or groups of samples, incorporating changes in expression of all parameters in high-dimensional space.

Preprocessing

Flow cytometry Standard (.FCS) files are data tables, where the rows are individual events and the columns are fluorescence (or light scatter) parameters, expressed in arbitrary channel units (fluorescence or light scatter intensity). Each fluorescence parameter represents a dimension in high-dimensional space. Together, their values describe the location of an event in high-dimensional space

The first step in preprocessing is to apply spectral compensation to the raw data. Spectral compensation matrices are often included in the header of the .FCS file.

The Pipeline

The phenotype is defined for each population of interest and recorded in a Marker Expression Table. An simple example is provided (Table). In our application, the phenotypic characteristics were user-defined based on knowledge of the biology and follow from the gating logic used to define them. Alternatively, phenotypes could be assigned by unsupervised population clustering, alone or in combination with expert knowledge. Parameters that are negative are encoded as 0, positive parameters can be 1 or 2 (dim or bright). Parameters that could be either positive or negative on a particular population are encoded as a "Don't Care" (DC) term.

	Parameter					
Cell Type	CD45	CD3	CD4	CD8	CD19	CD5
T cell	2	1	DC	DC	0	1
CD4+ T cell	2	1	1	0	0	1
CD8+ T cell	2	1	0	2	0	1
B cell	2	0	0	0	1	DC

Defining the Hamming Distance and Creating a Graph Layout

For each population pair, their Hamming Distance is calculated from the marker expression table, where distance equals the sum of differences of two populations with respect to the number of positions (columns, representing marker expression table). DC terms contribute a Hamming value of +1 from other populations.

The Hamming Distances for every pair of cell populations (called the Adjacency Matrix in graph theory) defines the Edges (connections) of the graph.

The Hamming table is used to build a population layout using an iterative graphing algorithm. The goal is to place each cell population in 2D space while maintaining the Hamming distances between the phenotypes. This layout will be used to visualize all data files with the same panel of markers.

Data Processing

Data annotation: Each event falling within a conventional analytical gate is annotated with a population identifier. Alternatively, events could be annotated as belonging to populations detected by an unsupervised clustering algorithm or semi-supervised classification.

Node Definition: Nodes consist of all events in the .FCS file categorized by the same annotation. As in conventional analysis, the proportion of events in each node is determined as the percent of total events or of events in the parent node. The node size (radius) in the graph is proportional to the percent of events in that node.

Node Connections: All node-pairs have a Hamming distance and could, in theory, be connected on the graph. The phenotype-aware nature of our application requires the user to determine which nodes are connected. For example, CD4+ and CD8+ nodes would be connected to a CD3+ parent node, but not to a CD19+ node. Thus, the user determines which Edges (connections) between nodes are shown on the graph. This step is guided by biological relationships and, once determined, is held constant for all samples analyzed with the same marker panel. Alternatively, node connections could be determined using a Minimum Spanning Tree, defined as the connections that leave no unconnected node and have the smallest sum of Hamming Distances.

Sinkhorn Distance: The Sinkhorn distance is calculated for every pair of events located in two populations that are connected by an Edge. This calculation requires the *location* of each event in multidimensional space (all fluorescence parameters) and the *Mass of Transport*. Initially a uniform "mass" is assigned for each event.

Euclidean Distance: The Euclidean distance is determined for every pair of events occurring in different nodes (populations) as defined by the annotations. This distance is known as the *cost of transport* for each event pair.

Optimal Transport Framework: The Optimal Transport Framework is a plan for moving the mass of transport between cell populations while minimizing the total *cost of transport* (sum of mass times cost for *n* event pairs). The *mass* of an individual event can be distributed among events (mass is initially uniform, but can be weighted by unequal distribution between events). Historically, this method worked for literally determining the most efficient way to move masses of earth (for building fortifications), but it is computationally intractable for high dimensional data.

The Sinkhorn distance solves this problem by providing an approximation of fundamental Optimal Transport. It uses the Sinkhorn matrix scaling algorithm:

Regularizer (lambda): An approximation parameter where larger values get closer to the actual optimal transport but lower lambda are less computationally intensive. Practically, lambda values between 0.1 to 0.5 work well in our application.

The Sinkhorn distance is computed for all population pairs (not just the ones in the graph).

In our current graph layout, Sinkhorn distance is encoded in the width of the edge connecting two populations (shorter distance = thicker edge).

Comparing two graphs

The final goal of this OT application is to compare graphs representing samples. Graphs are compared using a metric known as the Graph Edit Distance (GED). In comparing two graphs, three operations are possible for both nodes (populations) and edges (connections): Substitution, Insertion and Deletion.

GED counts every change as 1. The *cost* of a node operation is the change in percentage of the cell population. The *cost* of an edge operation is the change (difference) in Sinkhorn distance. The cost is calculated for all populations connected by edges on our graph.

Since node differences and Sinkhorn distance differences are on different scales, the node operations and edge operations are weighted in the calculation of the GED.
